# Game Plan: Development of a Web App Designed to Help Men Who Have Sex With Men Reduce Their HIV Risk and Alcohol Use

**DOI:** 10.2196/10125

**Published:** 2018-08-23

**Authors:** Tyler Wray, Christopher W Kahler, Erik M Simpanen, Don Operario

**Affiliations:** ^1^ Department of Behavioral and Social Sciences Brown University School of Public Health Providence, RI United States

**Keywords:** alcohol, HIV risk, internet, intervention

## Abstract

**Background:**

Men who have sex with men (MSM) are at high risk for HIV, and alcohol use is a major risk factor for HIV infection. Internet-facilitated brief interventions have been shown to reduce alcohol use and HIV-risk behavior in other at-risk populations, but have so far incorporated limited content and have not been tested among MSM.

**Objective:**

This manuscript describes Game Plan, an interactive, tablet-optimized web application designed to help heavy drinking, high-risk MSM consider reducing their alcohol use and sexual risk behavior. In this paper, we discuss the rationale, goals, and flow for each of Game Plan’s components, which were modelled after common in-person and web-based brief motivational interventions for these behaviors.

**Methods:**

The development of Game Plan was informed by a thorough user-focused design research process that included (1) audits of existing interventions, (2) focus groups with stakeholders and (3) intended users (high-risk, heavy drinking MSM), and (4) usability testing. The aesthetic, features, and content of the app were designed iteratively throughout this process

**Results:**

The fully-functional Game Plan app provides (1) specific and personal feedback to users about their level of risk, (2) exercises to help prompt users to reflect on whether their current behavior aligns with other important life goals and values, and for those open to change, (3) exercises to help users understand factors that contribute to risk, and (4) a change planning module. In general, this flow was constructed to roughly align with the two phases described in early accounts of motivational interviewing (MI): (1) Content intended to elicit intrinsic motivation for change, and when/if sufficient motivation is present, (2) content intended to translate that motivation into specific goals and plans for change. This sequence first focuses on the user’s HIV risk behavior, followed by their alcohol use and the connection between the two. The app’s overall aesthetic (eg, branding, color palettes, icons/graphics) and its onboarding sequence was also designed to align with the “spirit” of MI by conveying respect for autonomy, open-mindedness (ie, avoiding judgment), and empathy.

**Conclusions:**

Should future research support its efficacy in facilitating behavior change, Game Plan could represent a wide-reaching and scalable tool that is well-suited for use in settings where delivering evidence-based, in-person interventions would be difficult or cost-prohibitive.

## Introduction

### Background

In the United States, new HIV infections have declined among most at-risk groups in recent years but remain stable among men who have sex with men (MSM) [1,2]. As a result, MSM account for an ever-increasing percentage of all new infections, at nearly 67% in 2015 [3]. Alcohol use is a major risk factor for HIV infection, both among MSM and in other risk groups [4,5], and evidence suggests that this may be due in part to alcohol’s role in increasing the likelihood of engaging in sex that could transmit HIV. Specifically, event-level studies show that MSM are much more likely to engage in anal sex without protection after consuming 5 or more drinks on a given occasion [6,7]. This level of drinking is widespread among MSM. Nationally representative surveys suggest that 20.4% to 50.4% of young MSM drank at this level at least once in the last month [8,9]. Together, these studies suggest that exploring ways to reduce the spread of HIV among MSM is a critical and urgent public health priority [10,11] and that addressing alcohol use among these men may be a key target in this effort.

### Brief Motivational Interventions Can Change Alcohol Use and HIV Risk Behavior

Meta-analyses have shown that brief (30- to 60-minute) face-to-face interventions for alcohol use can help recipients reduce their drinking and these brief interventions are often as effective as longer and more extensive ones [12-14]. In particular, brief interventions that are inspired by the principles of motivational interviewing (MI) and use similar techniques (or brief motivational interventions [BMIs]) have received some of the most robust empirical support [12,15,16]. These interventions typically involve a handful of essential elements including feedback about recipients’ personal risk, emphasizing personal responsibility for change, providing a menu of change options, and enhancing self-efficacy for change, all of which should be conveyed with empathy [13]. BMIs have been shown to reduce heavy drinking and alcohol-related harms across a variety of key populations, including college students, treatment-seeking adults, and non–treatment-seeking adults [12,14,17]. Several recent studies have also shown greater reductions in alcohol use among heavy drinkers in HIV primary care settings who received a BMI compared with brief education and advice [18,19].

MI-inspired interventions (both brief and longer versions) focusing specifically on HIV risk behavior have also been shown to reduce these behaviors in a number of populations, including heterosexual HIV-negative and HIV-positive young adults [20,21], racial minority MSM [22], and people living with HIV in international settings [23]. Monti and colleagues [24] recently showed that a dual-behavior BMI reduced heavy drinking and condomless sex among heterosexual male and female emergency department patients compared with brief advice over 9 months of follow-up. Together, these results demonstrate that BMIs can help at-risk individuals change their alcohol use and sexual risk behavior and suggest that addressing both could provide additional benefit. However, to date, use of BMIs in many medical settings has been limited, perhaps because of high cost and resources needed to implement them. Delivering a similar intervention over the internet could ultimately provide a more scalable solution that is more likely to reach those at high risk in these settings, and therefore, could ultimately have a greater impact on public health.

### Brief Interventions Delivered Over the Internet Can Help Change Alcohol Use and HIV Risk Behavior

Given their brevity, BMIs have frequently been adapted for digital and Web-based delivery using a self-guided format [25]. Meta-analyses similarly support the efficacy of these Web-based BMIs for reducing alcohol use and related outcomes across a variety of settings and populations [26-28], with some evidence suggesting that they may be particularly successful among those who are especially high risk, like college students and heavy drinking medical clinic patients [26,29-31]. Few studies are available on similar interventions for HIV risk behavior, but at least 1 study has shown that an MI-inspired, internet-facilitated intervention increased condom use among heterosexual men and women [32].

One key limitation of existing Web-based BMIs, however, is that they have often incorporated only a small subset of the content that is typically included in face-to-face BMIs, and at least some of this content may be important in producing change. For example, most Web-based BMIs primarily involve developing discrepancy by providing basic feedback about behavior, but face-to-face MI interventions often incorporate a number of other approaches to building discrepancy, especially when clients are resistant to change [33]. These approaches frequently involve using activities or thought experiments to help develop discrepancy between clients’ current behavior and important life goals and values, such as the “looking forward/looking back” and “personal values card sort” exercises [34,35]. Building further discrepancy among clients who are resistant to change with these activities may be an important step in facilitating change [36,37].

Past research also shows that the strength of patients’ commitment to change is predictive of intervention outcomes in MI sessions [19,38,39]. Miller and Rose [40] argue that the explicit verbalization or acknowledgement of this commitment may not be a necessary precondition for change, but MI interventions involve tasks like change planning that are intended to help strengthen clients’ commitment to change. This involves presenting clients with a menu of potential change goals and allowing them to select specific steps they might take toward the goals they choose. While several existing Web-based BMIs offer users a list of strategies for reducing harm, few have incorporated substantive change planning components [28]. Addressing these limitations by integrating additional reflective activities that may further amplify discrepancy and incorporating substantive change planning components that may strengthen users’ commitment to change could help improve the efficacy of existing BMIs and lead to more durable changes in behavior [41].

### HIV Testing May Be an Opportune Time to Intervene

Past research on BMIs has shown that delivering these interventions at the right time could also boost their effects on health risk behaviors. That is, the impacts of BMIs may be most profound when delivered immediately after recipients have experienced negative consequences from a behavior because it may naturally prompt them to consider changing the behavior to avoid similar occurrences in the future [42,43]. With respect to alcohol use, meta-analyses have shown that college students who receive a BMI after an alcohol-related incident (an event that required medical, police, or administrative attention such as an injury, citation, or policy violation) showed greater reductions in drinking than wait-list controls [44] or those who received an abbreviated feedback intervention [45]. However, other studies have found few differences in alcohol outcomes across individuals who received BMIs after an incident or injury compared with heavy drinkers who did not experience these events [46-48].

With respect to HIV, in principle getting tested should constitute a part of routine health care for many high-risk MSM. However, in practice voluntary testing is often sought after a possible sexual exposure to HIV [49,50]. In 1 nationally representative sample, up to 60% of MSM listed a possible exposure as a reason for testing [51]. For those who test negative, having been potentially exposed to a life-threatening illness could naturally prompt MSM to consider the potential for change. As such, HIV testing could represent an optimal time to intervene because of this natural opportunity to initiate behavior change. Counseling is already routinely offered alongside voluntary HIV testing in many settings. There are standard recommendations about the content of this counseling [52], which involves providing basic education about HIV infection and identifying specific behaviors that may increase risk. However, in practice no specific approach to counseling is used consistently [53]. Moreover, in a recent study, Metsch et al [54] showed that a broad, person-centered counseling approach delivered alongside voluntary testing did not reduce sexually transmitted infection (STI) incidence or sexual risk behavior among MSM compared to information alone. While these findings have widely been interpreted as evidence that providing counseling alongside testing does not reduce risk, they could instead suggest that for posttest counseling to be effective, it must involve a more consistent, theoretically informed, and empirically supported approach to behavior change. Due to the urgent need to expand HIV testing and eliminate barriers [55], testing is also often provided by paraprofessionals who are given minimal training and guidance about counseling [56]. While this approach may indeed ensure that as many MSM who are at-risk have access to testing as possible, it may do little in terms of reducing future risk. Meanwhile, providing these paraprofessionals with thorough training in a specific counseling approach would be difficult and costly to implement, scale, and maintain [57]. Together, this evidence suggests that while voluntary HIV testing interactions may be a unique opportunity for individuals to reflect on their risk, consolidate motivation to reduce it, and plan for change, ensuring that those who currently provide testing are trained in empirically supported counseling techniques is likely prohibitive. Web-based approaches that provide many of these same components, however, may help capitalize on these opportunities without the need for extensive training and supervision.

### Goals of the Research

Given this landscape, we developed Game Plan, an interactive Web app optimized for tablet computers that aims to help high-risk MSM consider reducing their HIV risk behavior and alcohol use after they test negative for HIV at a clinic. Game Plan was designed to help users understand and reduce these health risk behaviors by (1) providing specific and personal feedback about their level of risk, (2) prompting them to reflect on whether their current behaviors align with other important life goals and values, and for those open to change, (3) helping them understand factors that contribute to risk and (4) making a plan to reduce risk behaviors in the future. This manuscript describes the components and their rationale. In this paper, we will provide an abbreviated overview of our methods for designing and developing Game Plan as well as its components and plans for future research to examine its efficacy in changing behavior.

## Methods

**Figure 1:**
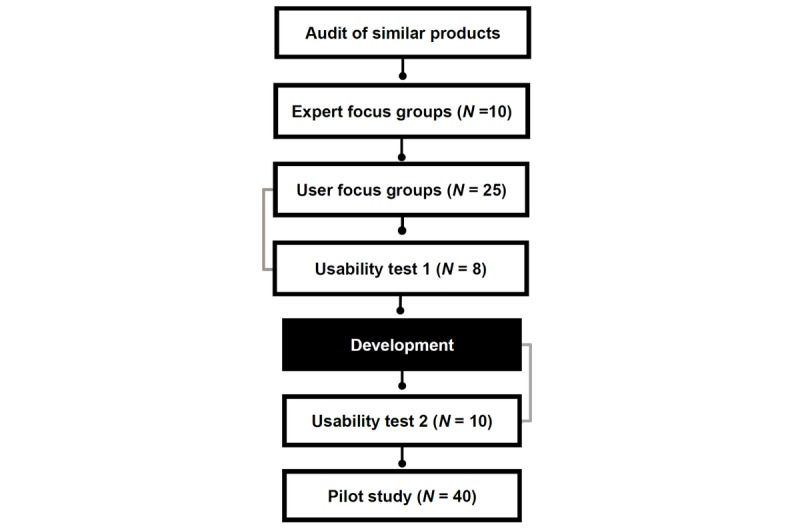
Design research phases and steps.

We began the development of Game Plan by conducting a thorough user-focused design research process [58,59]. The first step of this process involved conducting an audit of similar existing interventions and tools to compile lists of possible modules and their content. Next, we conducted a focus group with 10 subject matter experts and key informants (ie, HIV test clinicians and counselors) to better understand key content and the logistics of delivery in HIV testing clinics (eg, timing, duration). Then, we conducted participatory design focus groups with 25 high-risk, heavy drinking MSM who had recently tested for HIV. These groups directly engaged intended users in the process of design by enlisting their help in designing user personas, detailed, personal models that represent who the typical users of the app might be. These personas help the design team to develop empathy for the app’s intended users and provide them with a guide that can inform their decisions about the app’s design, flow, and aesthetic [58]. These steps can help designers create an app that is engaging and interesting to its intended users (see Figure 1).

## Results

### App Flow and Content

**Figure 2:**
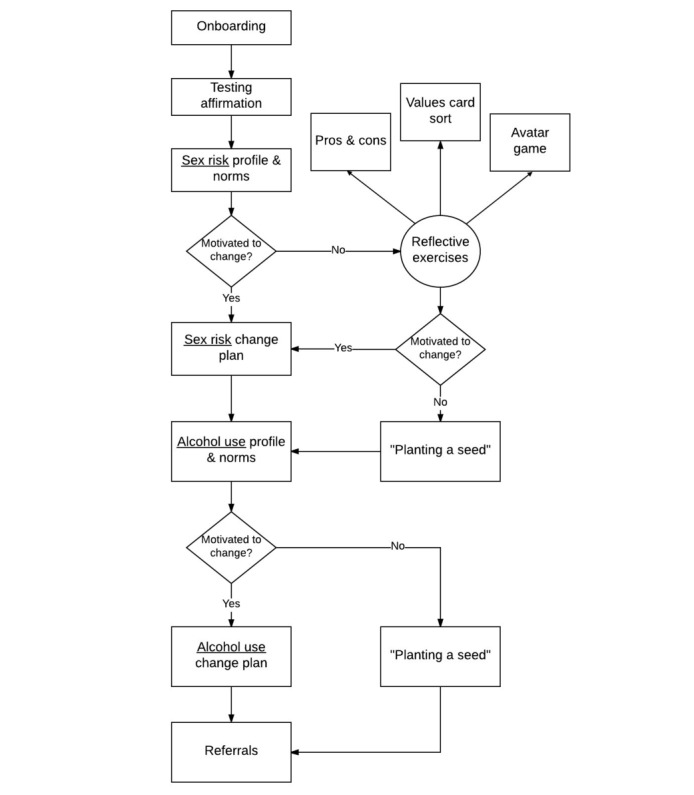
Flow of Game Plan components.

The following sections review the overall spirit we hoped to achieve in the fully developed Game Plan app as well as each of its components. Table 1 presents these components and a brief summary of their purpose, and a flow of these components is shown in Figure 2. In general, this flow was constructed to roughly align with the 2 phases described in early accounts of MI: content intended to elicit intrinsic motivation for change, and when/if sufficient motivation is present, content intended to translate that motivation into specific goals and plans for change. This sequence first focuses on the user’s HIV risk behavior followed by their alcohol use and the connection between the two.

### Overall Spirit, Aesthetic, and Onboarding

**Figure 3:**
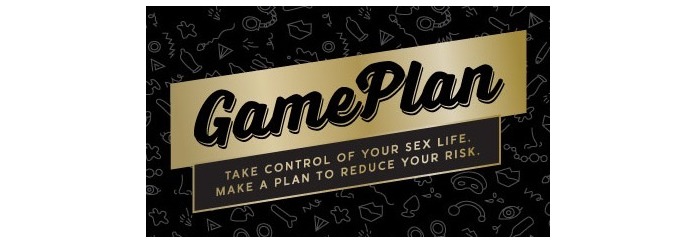
Game Plan branding.

Past research on MI shows that the therapist’s interpersonal style is one of the strongest predictors of change [60,61]. This style calls for therapists to respect the client’s autonomy and freedom to choose, be collaborative, avoid judgment, and elicit the client’s own motivation rather than trying to impart it. Empathy, or developing a thorough understanding of another’s perspective and reflecting that understanding back, is at the heart of this style [60]. In Game Plan, we attempted to convey a similar style by including 3 screens in the onboarding process that explicitly informed them of this purpose. This mirrors what many MI therapists do at the beginning of their sessions [33] and is an approach adopted by many other digital behavior change interventions [28]. We also attempted to convey this style by designing the app’s aesthetic to reflect open-mindedness (ie, avoiding judgment) and respect for autonomy. To facilitate this, designers were briefed on the “MI spirit” and attended user focus groups in person, ultimately developing the app’s aesthetic (eg, branding, color palettes, icons, and graphics) with this style in mind. To help convey a nonjudgmental and open tone, we believed it was important to adopt a sex-positive, relatable, and at times, playful, aesthetic. Yet, given Game Plan’s purpose of providing honest information about risks, it was important that these concepts be reflected in a classy and refined way. For these reasons, the direction we chose incorporated playful, handwritten icons to convey sex-positivity, relatability, and respect for users’ choices and a color palette intended to reflect a classy tone while ensuring readability. This aesthetic is represented in Figure 3.

### Affirming HIV Testing

Since Game Plan was developed specifically for use in HIV testing situations, we believed that first acknowledging users’ feelings and thoughts about testing could be an important way to communicate empathy and transition them to the task of reflecting on their sexual choices. Thus, after users complete the onboarding sequence, 2 screens ask users to indicate how they are feeling and what they are thinking about testing for HIV. Users drag various feelings and thoughts into an area represented by a heart or thought bubble. A modal dialog window then appears providing an empathetic message tailored to their responses.

**Table 1:** Brief summary of Game Plan components.

Section		Goals
Testing affirmation		Help users express feelings and thoughts about testing Express empathy
Demographic assessment		Assess fit for the users (eg, high-risk men who have sex with men [MSM]) Assess tailoring variables (eg, age)
**HIV risk**		
	Assessment	Assess number of partners and their HIV statuses in the past year Assess number of insertive/receptive anal sex acts with and without condoms or preexposure prophylaxis with status unknown partners
	Profile and norms	Present potential risk for HIV in the past year, compare to overall risk in men, MSM Compare number of partners, condomless anal sex events in the past year with other MSM
	Reflective activities	Weigh the pros and cons of changing choices about sex Reflect on how sexual choices affect future goals Reflect on how well sexual decisions align with important personal values
	Change planning	Explore menu of options for reducing risk Identify important reasons for making that change Identify the specific steps involved in making change Identify a specific start date for each change
**Alcohol use**		
	Assessment	Assess number of drinking days in the past 30 days Assess quantity of drinking on each day
	Profile and norms	Present feedback for alcohol risk, binge drinking, and typical quantity Compare drinking with other MSM
	Alcohol and HIV risk	Review risks involved in sex under the influence Assess reasons for drinking prior to or during sex Reframing beliefs about alcohol’s effects on sex
	Change planning	Explore menu of options for changing alcohol use or alcohol-related harm Identify specific steps to make changes in drinking Identify a specific start date for this change and/or others who can help
Referrals		Present contact information and hours for various medical and mental health services Allow users to print or email their change plan anonymously

### Demographic Assessment

For program purposes, the next section assesses users’ basic demographic characteristics including age, sex assigned at birth, current biological sex, current gender, current sexual orientation, preexposure prophylaxis (PrEP) use, and region of residence in the United States. To ensure anonymity of users, no other personal or identifiable information is collected.

### HIV Risk Assessment, Profile, and Norms

**Figure 4:**
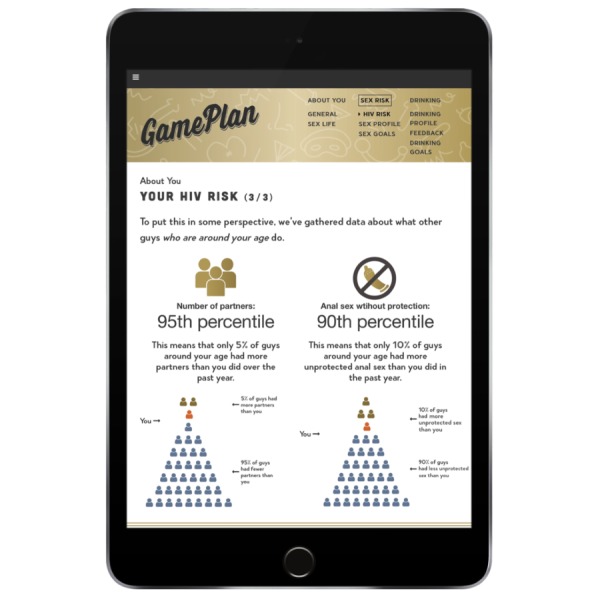
Example screen providing feedback on HIV risk behavior.

This section provides personal, easy-to-understand estimates of users’ potential risk for HIV in the past year given their current sexual behavior during that time and their projected 5-year risk using data from past research [62-65]. It also compares users’ total number of sex partners and number of times they engaged in anal sex without a condom in the past year with other gay and bisexual men in their age group using available national survey data [66]. The purpose of this feedback is to help develop discrepancy between users’ recent behavior and what they would like it to be in the future, a central theme of MI [37]. To inform this feedback, users first complete an assessment of their risk behaviors over the past year (ie, number of insertive/receptive anal sex acts with unknown status partners without using condoms or either partner using PrEP). Reminders about the app’s anonymity and nonjudgmental approach are provided in this section to encourage honesty. An example of this screen is presented in Figure 4.

### Reflective Activities

After the final feedback screen, a modal dialog asks users about their current motivation to start making safer choices about sex. Users rate this scale from 1 (not at all) to 9 (a lot), and those who select a value less than 7 are directed to a landing page that presents 3 exercises designed to help encourage users to reflect further on their sex lives and whether their recent choices align with their broader goals and values. Each of these activities was modeled on similar thought exercises commonly used in MI intended to elicit change talk or client speech that provides arguments for change [35]. While Game Plan’s reliance on a tablet-based drag-and-tap interface clearly does not permit users to verbalize these motivations through speech as they would in face-to-face MI sessions, we believe that prompting users to reflect through these activities may encourage a similar internal process that underlies change talk. The first of these is a pros and cons exercise and is similar to decisional balance exercises used in a number of in-person and internet-delivered personalized feedback interventions (PFIs) [67-69]. It is designed to help users identify both the positive aspects of their recent sexual decisions and the drawbacks of these choices and determine whether the drawbacks ultimately outweigh the positives. The second is a values card sort and is very similar to a card sort often used in face-to-face MI sessions [34]. Users are presented with a deck of cards on which several personal values appear and asked to select 3 cards that best represent values that are important to them. Users are then presented with feedback about their sexual risk behavior from earlier sections and asked to rate how consistent these recent sexual choices are with their selected values before being provided with a tailored dialog message reflecting this response. Finally, the avatar exercise is intended to encourage users to picture what they hope their lives will be like in 10 years and reflect on whether their recent sexual decisions will get them closer to or further away from those goals. Users construct an avatar reflecting their future selves and are asked to identify goals they hope to achieve in the next 10 years. Feedback about users’ sexual risk behavior in the past year from earlier sections is then presented. Users are asked to imagine they made changes to be safer and rate whether that might get them closer to or further away from these hopes on the same scale. Once users complete at least 2 of these exercises, a dialog window appears asking them to again rate how much they would like to start making safer sexual choices on a 1 (not at all) to 9 (a lot) scale. Those responding with a 2 or above (not really) are directed to a change planning section that presents several options for reducing their sexual risk. Those who rate this item a 1 (not at all) are directed to a section intended to plant a seed for changing their sexual risk behavior in the future.

### Change Planning to Reduce Sexual Risk

**Figure 5:**
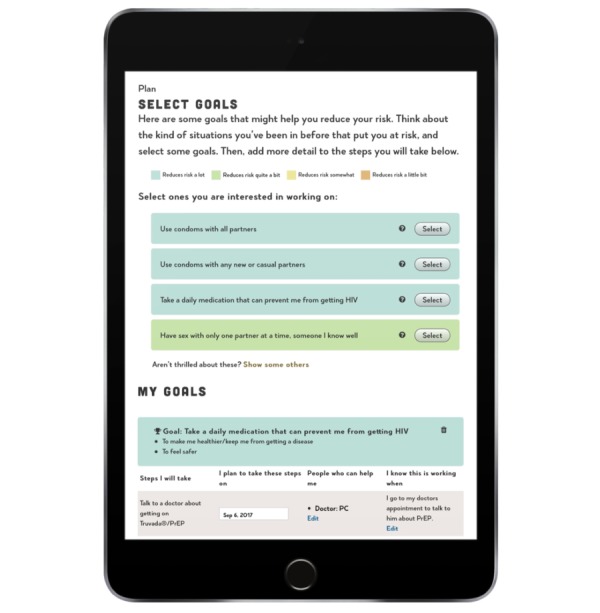
Example change planning screen for HIV risk behavior. PrEP: preexposure prophylaxis.

The sex risk change planning section presents users with a menu of possible goals that could help them reduce their risk and assists them in identifying steps they can take toward those goals. Goal options are shown and color coded according to the extent to which they reduce risk for HIV, with those that reduce risk a lot presented in green, those that reduce risk a little bit presented in yellow, and those that reduce risk somewhat in orange and red (see Figure 5). Those that reduce risk a lot are presented on the first screen, but users who are not satisfied with these options can click a link to reveal those that reduce risk somewhat or a little bit. Once a goal is selected, users can identify important reasons for making this change, practical steps that could help them, and when they will start. Doing so adds this information to a change plan that appears below and can be later printed or emailed to themselves.

Those who rate that they are not at all interested in making a change are directed to an exercise about planting a seed commonly used in MI for those low in motivation to change [33]. This section was included given evidence from past research that pushing these users to begin planning for change may further increase their resistance and decrease the likelihood of change [38]. This section first provides a summary (eg, “it sounds like you’re pretty happy with your sex life as it is now”) before asking users to identify specific events that, if they occurred, might make them think about making a change to be a little safer.

### Alcohol Use Assessment, Profile, and Norms

After these sections, a dialog box appears that transitions the focus to alcohol use. The alcohol use assessment and feedback screens are very similar to many other existing PFI tools [25,28]. After completing an assessment of drinking in the last 30 days using a graduated frequency approach [70,71], users’ drinking is classified as moderate or hazardous according to National Institute on Alcohol Abuse and Alcoholism guidelines [72]. The total number of drinks consumed, total binge drinking days, and average number of drinks per drinking day are then shown before comparing users’ drinking with that of other gay and bisexual men in the United States [73].

Other screens in this section focus on the connections between alcohol use and sexual risk, noting the role of drinking in HIV transmission [5,7] and asking users to identify specific reasons they might drink prior to or during sex [74]. Brief text content is then presented addressing the specific reasons users identified, suggesting an alternative interpretation of alcohol’s effects on sexual behavior based on available research. Finally, users assess their motivation to change their drinking in a manner similar to earlier sections. Those indicating an interest in changing are directed to an alcohol-specific change planning section, while those decidedly resistant are directed to a section intended to plant a seed for changing their drinking in the future.

### Change Planning for Alcohol Use

The alcohol change planning and planting a seed sections are similar to those used for sex risk. In these sections, users select from drinking goals (ie, stop drinking entirely, reduce how much I’m drinking or cut down, or change how I’m drinking to keep bad things from happening as often), which are similarly color coded. Users can indicate important reasons for making this change, specific steps they can take (including protective behavioral strategies [75,76]), and a date they will start. These goals and their details are then added to any other goals they had selected throughout their session. Afterward, all users are directed to a final page where they can elect to either print or email their plan and/or information on referrals to themselves. Referral information is provided for local lesbian, gay, bisexual, and transgender–friendly agencies and organizations that provide HIV/STI testing, general medical care, mental health care, and alcohol and drug treatment. To ensure users’ anonymity, no personal information is included in these materials and a portable printer is used for those who elect to print their materials.

## Discussion

### Principal Findings

To our knowledge, Game Plan is one of the first internet-facilitated, combined brief interventions that focuses on both HIV risk behavior and alcohol use. The features described in this paper also set it apart as among one of the most complete self-guided brief interventions inspired by the principles of MI. Meta-analyses exploring the effects of digital and internet-facilitated behavioral interventions provide strong evidence that highly tailored interventions may be more successful in facilitating behavior change than those that present all users with similar content [25,77,78]. Game Plan provides content that is tailored to users’ demographic characteristics (eg, age, sexual orientation), behavior (eg, sexual risk, alcohol use), readiness to change, preferred change goals, and other characteristics. The overall spirit, structure, and aesthetic elements of Game Plan were also designed specifically with the needs of intended users (high-risk, heavy drinking MSM) as a roadmap. To facilitate this, we used interaction design research methods, a process used in many industry software development projects [58,59]. This process enabled all members of our design and development team to thoroughly understand the motivations that intended users might have for engaging with this tool so that we could use these insights to help guide our design decisions.

Game Plan was specifically designed to be used alongside HIV testing, when intended users may already be naturally considering behavior change and when prevention services reach many intended users. Offering Game Plan to users after HIV test results have been provided may also be fitting because its content addresses many of the same topics that are often priorities of traditional posttest counseling including identifying and assessing risk behaviors and developing a plan to reduce risks [52]. However, Game Plan may approach these tasks in a manner that is more consistent with existing evidence-based interventions that have been shown to reduce HIV risk behavior and major precipitating factors (ie, alcohol use [24,27]). Given that guidelines on HIV testing and counseling recommend that prevention counseling not be a required component of HIV testing, it may be that Game Plan could best be used as an alternative for those who are uncomfortable with or are unwilling to engage in traditional counseling or as a complement to it.

### Future Directions

Research on Game Plan is currently in its preliminary phases. As such, little is known about its effects on behavior change, including when and how it might be most effective or how it compares with other available internet-facilitated brief interventions and comparable evidence-based face-to-face interventions. While a pilot test of its effects on behavior and theoretical mediators of change (eg, readiness to change, self-efficacy, norms) is currently ongoing, a larger trial will be needed to explore whether it helps intended users change their behavior and whether these changes, in turn, impact key end points like HIV/STI incidence. If evidence supports its efficacy, future research could also be devoted to optimizing the efficacy of its components. Finally, if Game Plan’s effects appear promising, further research will also be needed to inform implementation and dissemination strategies, including a plan for providing ongoing technical maintenance, support, and updates.

### Limitations

While Game Plan has many strengths, several limitations are also important to note. First, Game Plan’s components were developed based on existing models of brief interventions. As such, it was intended to provide users with only a single exposure to intervention content and provides no longer term support for behavior change over time. More frequent support will likely be needed in order to achieve durable changes in both HIV risk behaviors and alcohol use. This could involve expanding this program to allow users to check in on their goals over time or by using existing text message interventions for these behaviors [79,80]. Similarly, brief interventions that served as models for Game Plan were based on the principles of MI, which is a person-centered counseling technique. As such, these approaches emphasize that therapist characteristics like warmth, genuineness, and empathy [60] are key to successfully facilitating behavior change. Some of these characteristics are most intuitively conveyed through natural language and other, nonverbal behaviors (eg, by mimicking another’s expressions of emotion [81]), and some concepts like empathy may only be conveyed by a human interventionist, since they often involve a person’s ability to subjectively experience and understand another person’s feelings [82]. As such, the point-and-drag interface used by Game Plan may be less natural or impactful than the interactions provided in traditional face-to-face MIs, since it lacks these important interpersonal elements and could also fail to incorporate critical facets of MI interventions that lead to change. However, our past research on brief computer-delivered interventions for alcohol use has shown that participants rate both natural language and point-and-click interfaces as consistent with an MI counseling style. Moreover, there were no differences between participants assigned to interact with the intervention via these 2 interfaces, either in terms of their willingness to set a goal or in their alcohol use a month after intervention [83]. A final limitation is that Game Plan was designed specifically to meet the needs of high-risk gay, bisexual, and other MSM. Given that content focuses on anal sex with other MSM, its use should be limited to this population.

### Conclusion

Game Plan is a brief, tablet-optimized intervention that aims to help gay, bisexual, and other MSM reduce their alcohol use and risk for HIV by providing interactive, personal feedback about users’ risks, facilitating reflection on the impact that these risks have on users’ lives and goals and enabling them to select from a menu of possible change options to begin planning for that change. Developed to mirror common components of existing evidence-based brief interventions for these behaviors, Game Plan could represent a wide-reaching and scalable tool that can help facilitate risk-reducing behavioral changes in settings where delivering evidence-based in-person interventions would be difficult or cost prohibitive.

## Abbreviations

BMI: brief motivational intervention
MI: motivational interviewing
MSM: men who have sex with men
PFI: personalized feedback intervention
PrEP: preexposure prophylaxis
STI: sexually transmitted infection
